# Knowledge, attitude and perception towards COVID-19 vaccines amongst clients of tertiary care hospital in Dar es Salaam, Tanzania

**DOI:** 10.11604/pamj.2024.49.114.39559

**Published:** 2024-12-10

**Authors:** Mohamed Zahir Alimohamed, Shahista Jaffer, Gibson Kagaruki, Anna Jazza, David Andimile, Kaushik Ramaiya

**Affiliations:** 1 Research and Training Department, Shree Hindu Mandal Hospital, P. O. Box 581, Dar es Salaam, Tanzania,; 2 Department of Haematology and Blood Transfusion, Muhimbili University of Health and Allied Sciences, P.O. Box 65001, Dar es Salaam, Tanzania,; 3 Department of Biochemistry and Molecular Biology, Muhimbili University of Health and Allied Sciences, P.O. Box 65001, Dar es Salaam, Tanzania,; 4 Tanzania Human Genetics Organization, Dar es Salaam, Tanzania,; 5 Department of Genetics, University Medical Center Groningen, University of Groningen, Groningen, the Netherlands,; 6 National Institute for Medical Research, Tukuyu Research Center, P.O. Box 538, Tukuyu, Tanzania

**Keywords:** Knowledge, attitude, perception, COVID-19 vaccine, vaccine-seeking communities, non-vaccine-seeking communities

## Abstract

**Introduction:**

COVID-19 vaccine uptake has been poor around the globe due to various reasons, including misperception about disease and vaccines due to fabricated news amidst social platforms and personal beliefs to name a few. We aimed to assess the knowledge, attitudes, and perceptions of people attending our institution regarding these vaccines.

**Methods:**

a hospital-based cross-sectional study was conducted at Shree Hindu Mandal Hospital in August 2021. These communities were patients attending the medical departments at the hospital. Bloom technique was used to grade individuals’ attitudes, perceptions, and knowledge levels towards COVID-19 vaccines. Association between the explanatory variables and low knowledge, negative perception, and negative attitudes towards vaccines were assessed using a T-test, Chi-Square, and modified poison logistic regression model.

**Results:**

this assessment involved 1547 communities (vaccine seeking community=547 and non-vaccine seeking community=1000). A high level of vaccine awareness 99.3% (n=1536) was observed. Low knowledge, negative perception, and negative attitude toward COVID-19 vaccines were 49.1%, 28.5%, and 30.1% respectively. Medical care services seekers were more likely to have low knowledge as compared to the counterpart aPR=1.6(95% CI: 1.4 -1.8), those who did not get vaccine information from social media aPR=0.89(95% CI: 0.81-0.99) and social gatherings aPR=0.80(95% CI: 0.75-0.94) were less likely to have low knowledge as compared to those who received the vaccine information from those sources. Medical care services seekers were more likely to have a negative perception towards vaccines as compared to vaccine seekers aPR=4.8(95% CI: 3.4-6.7), those who did not get information from social media aPR=0.80(95% CI: 0.70-0.90) and neighbor/friends aPR=0.82(95%CI: 0.70-0.96) were less likely to have a negative perception towards vaccines. Medical care services seekers aPR=0.5(95% CI: 0.4-0.6) were less likely to have a negative attitude while those who received vaccine information from neighbors/friends aPR=1.5(95% CI: 1.2-1.8) were more likely to be found with negative attitudes towards vaccines.

**Conclusion:**

low knowledge, negative attitude, and perception towards the COVID-19 vaccine were observed. Sources of vaccine information and being not a vaccine seeker play a significant role in the level of low knowledge, negative attitude, and perception. We recommend interventions to improve knowledge, attitude, and perception towards vaccines and modulation of sources of vaccine information for improved uptake of vaccines.

## Introduction

Over the past two years, the world has been battling the COVID-19 pandemic which is attributable to innumerable deaths globally. The World Health Organization (WHO) reports that 6,859,093 deaths have been confirmed due to COVID-19 since the pandemic began [1]. An important breakthrough was the discovery of COVID-19 vaccines which have proven to be effective in preventing severe forms of the disease, thus, reducing hospital mortality and morbidity rates.

Several effective vaccines have been developed using different approaches such as messenger RNA, this type of vaccine uses genetically engineered mRNA to give your cells instructions for making the S protein found on the surface of the COVID-19 virus. Others include a vector vaccine that uses genetic material from the COVID-19 virus, which is placed in a modified version of a different virus (viral vector), and a protein subunit vaccine that includes only the parts of a virus that best stimulate your immune system. This type of COVID-19 vaccine contains harmless S proteins [2]. Commonly known vaccines include Pfizer, AstraZeneca, Janssen, Moderna, and Sinopharm to name a few. Despite the availability and accessibility of these vaccines across continents, uptake has been poor and individuals are hesitant globally [3]. As of 27^th^ February 2023, 13.228 billion doses have been administered globally, and more than half of the world´s population (67.5%) is already vaccinated with one or more doses of a vaccine. Africa has been on a steady increase with 24% of its population vaccinated whether fully or partly [4].

Acceptance of vaccines is a limiting factor in achieving herd immunity. Herd immunity is a crucial outcome of vaccination programs where individuals who are vaccinated indirectly protect other people from getting the disease [5]. The more people that are vaccinated, the quicker herd immunity is achieved and the spread of disease halts. Several studies have determined reasons for the slow uptake of vaccines which include: poor knowledge, misguidance due to misinformation, and personal or traditional beliefs that they are immune to this disease amongst others [6-8]. A randomized trial with the intervention being exposure to misinformation about COVID-19 and the vaccines showed that misinformation led to poor acceptance of the vaccine [9]. Trust in the government is a crucial observation that has been associated with high acceptance rates in many countries [10]. Tanzania was amongst the countries that despite taking a stand on observing the precautionary measures in the first wave when vaccines were first announced, took a step back and asked for its internal evaluation before urging its people to take it [11,12]. This was so because the political leadership lacked clarity on its safety for the African population and a lot of messages that were dispersed around prevention of COVID-19 were mixed bringing about confusion amongst most individuals on the country´s status regarding COVID-19 vaccine use. With the change in the leadership, there has been a shift from “denial” to “acceptance” and the country has joined the COVEX program to make vaccines available [13].

In August 2021, Tanzania received a total of 1,609,400 vaccines via the COVID-19 Vaccines Global Access (COVAX) Facility as the first consignment [14]. Access to vaccines was severely limited in sub-Saharan Africa, including Tanzania, but the availability of vaccines has increased recently. Given this, we aimed to assess the knowledge, attitudes, and perceptions of people who came to our center for vaccination purposes and compare it to patients attending various outpatient departments within our center not seeking vaccines. We also aimed to estimate the extent, if any, of vaccine hesitancy.

## Methods

**Study setting:** the study took place at Shree Hindu Mandal Hospital (SHMH) in August 2021. Shree Hindu Mandal Hospital served as a vaccination site for the public; one among multiple appointed centers of vaccine distribution, offering Jansen & Jansen COVID-19 vaccines free of cost.

**Study participants and recruitment:** we had two study populations, those seeking medical care from various outpatient services and those seeking only vaccination services. All adult individuals who consented to respond to the questionnaire were included in this study and recruitment of study participants took place at the waiting rooms within the facilities. Pregnant women and children were excluded from the study.

**Study design:** this was a hospital-based cross-sectional study to assess the knowledge, attitude, and perceptions (KAP) on COVID-19 vaccines among clients attending outpatient services and those seeking vaccine facilities at SHMH during August 2021.

**Sample size and sampling procedures:** a formula for cross-sectional study design was used to calculate sample size [15]. In a study conducted in Malaysia, it was observed that 38% of the community had good knowledge of the COVID-19 vaccine [16]. We assumed a 5% level of significance, a 2.5% margin of error, and a 10% non-response rate after which our sample size was estimated to be 1609 community members. This sample size was distributed into two groups i.e., vaccination and medical care service seekers. Based on pilot data from the Outpatient Department at Shree Hindu Mandal Hospital, it was observed that about one-third of the community who visited the hospital during the period of the study were seeking the COVID-19 vaccine. Therefore, one-third (537) and two-thirds (1072) of the participants were envisaged to be vaccination and medical care services seekers respectively. However, our actual sample size was 1547 resulting in a response rate of 95.5%. Pre-service interview was conducted amongst both medical care and vaccination services seekers. All service seekers who come to SHMH are registered in the system before receiving the health services. The data manager who works at the outpatient department (OPD) was trained by the principal investigator of this project on the recruitment procedures of the study participants. After registration, a service seeker was asked to explain the purpose of visiting SHMH on that particular day. The reasons for visiting the facility were categorized into two categories either seeking medical care services or seeking vaccine services. In both study groups, the data manager selected one service seeker after every 10^th^ attendee until the required sample size was met i.e., systematic sampling was applied consecutively till the required sample size was reached. The research assistants were responsible for seeking informed consent from the participants.

**Data collection:** a structured questionnaire with closed and open-ended questions was designed and distributed to medical care and vaccination service seekers within SHMH. The same data collection tool was distributed to community members who were waiting for the COVID-19 vaccine service at the vaccination site. Both completed and non-filled questionnaires were collected from the respondents during exit time.

**Study variables:** this study had three outcome variables including knowledge, perception, and attitude toward the COVID-19 vaccine. For knowledge assessment, five items were used to assess knowledge on the COVID-19 vaccine, for which a score of “1” and “0” for a correct and incorrect response was given, respectively. For example, the question that asked: “Can the COVID-19 vaccine protect you from getting COVID-19?” Here the correct response was “yes” and the incorrect response was “no” or “don´t know”. Those who responded correctly to an item were considered as having adequate knowledge and vice versa. An individual´s scores were aggregated to generate an overall score which ranged from 0 (minimum) to 5 (maximum). Each individual´s overall score was converted into percentage scores using max scores (5) as the denominator. Later, bloom technique 1 was used to categorize individual´s scores into three: low, moderate, and high if one, scored <60% or 0-2 scores, between 60-79.9% or 3 scores, and 80-100% or 4-5 scores respectively. This variable was re-categorized again to generate binary responses i.e., low knowledge (low) and adequate knowledge (moderate or high). We also assessed the perception of participants on the COVID-19 vaccine using three questions, a score of “1” and “0” was given to each answer if the response was favoring the vaccine or not respectively. Individual scores were aggregated to generate an overall score which ranged from 0 (min) to 3 (max). Later, individual scores were categorized into three categories namely negative (0-1 scores), neutral (2-scores), and positive (3-scores). These categories were later converted into binary outcomes and categorized as follows: Negative (0-1) and neutral/positive (2-3). The last outcome assessed was attitude; this domain had three items like perception thus the process of grading the responses was the same as perception.

Twelve variables were used to explore the prediction of each of the three outcomes of interest i.e., low knowledge of the COVID-19 vaccine, negative attitude, and perception of the vaccine. Those explanatory variables were grouped into three groups namely demographic variables (gender, age, occupation, marital status, and education level). The second category was the source of information for the COVID-19 vaccine (1) official government and international websites and media; 2) news media e.g., TVs, radios, newspaper; 3) social media e.g., WhatsApp, Facebook, Instagram, and Twitter; 4) medical journals; 5) neighbors, friends, and family, and 6) social gatherings). The last group was the type of community (those receiving the vaccine versus those not seeking the vaccine). Setting base category was done as follows: sex (male vs female (referent)), age of respondent (18-30 years, 31-40 years, and 41-50 years and >50 years (referent)), occupation (unemployed, formal employment, student and retired (referent)), marital status (single, others and married (referent)) and education level (none/primary education, secondary education and college and above (referent)). All six sources of information for the COVID-19 vaccine had yes and no responses and yes was referent. For the type of community non-vaccine seekers were the reference/base category.

**Data analysis:** a comparative analysis was made to assess if there were significant differences between demographic characteristics such as age, setting, sex, education, marital status, occupation, and outcomes of interest regarding KAP for COVID-19 disease and vaccines. T-test was used to assess the mean difference of the continuous variables such as age and explanatory categorical variables. We also conducted a comparative analysis of binary outcome variables with categorical explanatory variables using the Chi-square test. Unadjusted and adjusted analysis using the Modified Poison Logistic Regression Model was used to assess factors associated with the outcome variables of interest; low knowledge and negative perception/attitude. All prevalence of outcomes were above 10% thus prevalence ratios (PR) were estimated using the Modified Poison Logistic Regression Model and we reported their 95%CI. All independent variables whose unadjusted prevalence ratio (aPR) variables were significant at p<0.20 were qualified for multivariable analysis. Furthermore, a variable was considered statistically significant in the multivariable model or adjusted analysis at a level of p<0.05, and this stage adjusted prevalence ratios (aPR) were reported.

**Informed consent:** participants were provided with informed consent before the questionnaire acknowledging their voluntary involvement in the study. Participants were informed about the purpose of the study, study procedures, potential risks involved, and their right to withdraw from the study at any time without consequences.

**Ethical clearance:** the ethical clearance was obtained on 8^th^ November 2021, Ref No: NIMR/HQ/R.8a/Vol.III/95 from the National Institute of Medical Research. The clearance affirmed that the study adhered to ethical principles and the rights, privacy, and confidentiality of the participants throughout the research process.

## Results

**Socio-demographic characteristics:** a total of 1547 participants were included in this study of which 547 (35%) attended the vaccination site and 1000 (65%) attended the outpatient department for medical care. Males (813) comprised 52.7% of the population. The mean age of the participants was 40.9 years (SD = 14.8). Participants who received the vaccine were mostly over the age of 50 years (193, 35.3%) while the participants who didn´t seek the vaccine were aged between 18 to 30 years (331, 33.2%). A total of 852 (55%) attendees were married and 879 (57.1%) had formal employment in both participant groups (Table 1).

**Table 1:** demographic characteristics of study participants (n=1547)

Variable	Category	Overall, n=1547 (%)	Non vaccine seekers n=547 (%)	Vaccine seekers n=1000 (%)	P-value
**Gender**	Male	813(52.7)	315(58.0)	498(49.8)	0.002
	Female	730(47.3)	228(42.0)	502(50.2)	
	Missing	5			
**Age**	**Mean (SD)**	40.9(14.8)	43.9(14.3)	39.2(14.8)	<0.001
	**Age group**				
	18-30yrs	450(29.1)	119(21.8)	331(33.2)	<0.001
	31-40yrs	395(25.6)	122(22.3)	273(27.4)	
	41-50yrs	306(19.8)	113(20.7)	193(19.3)	
	>50yrs	394(25.5)	193(35.3)	201(20.1)	
**Occupation**	Unemployed	341(22.2)	126(23.4)	215(21.5)	0.001
	Formal employment	879(57.1)	328(60.9)	551(55.1)	
	Student	163(10.6)	36(6.7)	127(12.7)	
	Retired	156(10.1)	49(9.1)	107(10.7)	
**Marital status**	Single	422(27.3)	121(22.2)	301(30.1)	<0.001
	Married	852(55.2)	380(69.7)	472(47.2)	
	Divorced	145(9.4)	15(2.8)	130(13.0)	
	Widow/widower	126(8.2)	29(5.3)	97(9.7)	
**Education level**	Never attended	85(5.5)	7(1.3)	7(7.8)	<0.001
	Primary education	303(19.6)	101(18.5)	202(20.2)	
	Secondary education	581(37.6)	161(29.4)	420(42.0)	
	College and above	578(37.4)	278(50.8)	300(30.0)	
**Ever heard COVID vaccine**	No	11(0.7)	0(0.0)	11(1.1)	0.014
	Yes	1536(99.3)	547(100)	989(98.9)	

**Figure 1:**
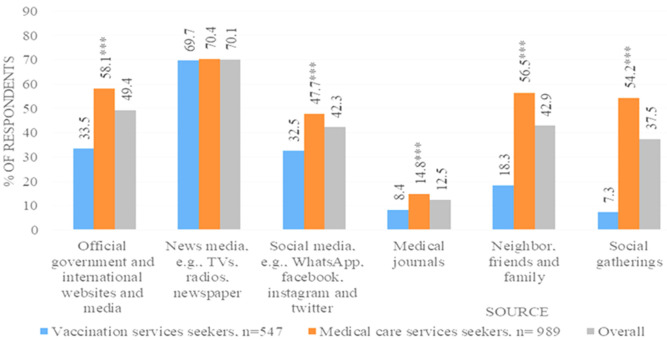
source of information for COVID-19 (n=1536)

Overall, all the participants with a high level of education and secondary level of education and a college degree were 581(37.6%) and 578(37.4%), respectively (Table 1). Most participants 70.1% cited the news media i.e., TV, radio, and newspaper as sources of information on COVID-19 vaccines. Participants who didn´t come to the hospital to seek the COVID-19 vaccine obtained information from all sources in higher proportions except the news media where both types of study participants were almost equal in proportion (Figure 1).

**Figure 2:**
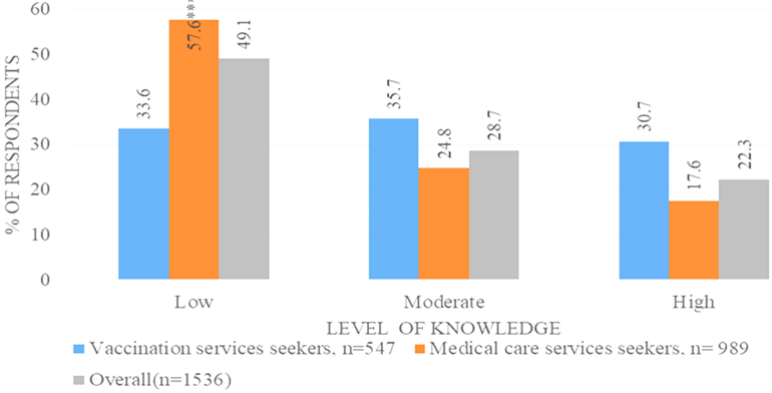
proportion of respondents with low, moderate, and high COVID-19 vaccine knowledge level by type of community (n=1536)

**Knowledge on the COVID-19 vaccine:** most respondents 1536 (99.3%) reported that they had heard of the COVID-19 vaccine. Both the participant groups who visited the hospital to seek COVID-19 vaccines and other services had low levels of knowledge (49.1%) (Figure 2). Official government and international websites, neighbors, friends and family, social gatherings, and social media showed significant association with low knowledge among the non-vaccinated participants (Figure 1). However, adjusted analysis results indicated that; medical care services seekers were more likely to have low knowledge as compared to the counterpart aPR=1.6(95%CI: 1.4 -1.8), those who did not get information from social media aPR= 0.89(95%CI: 0.81-0.99) and social gatherings aPR=0.80 (95%CI: 0.75-0.94) were less likely to have low knowledge as compared to those who received the information from those sources (Table 2).

**Table 2:** association between social demographic characteristics, source of COVID-19 vaccine information, type of community and negative perception, negative attitude, and low knowledge towards COVID-19 vaccine: unadjusted and adjusted modified poison logistic regression model

Variable	Negative perception		Low knowledge		Negative attitude	
	uPR, 95%CI	aPR, 95%CI	uPR, 95%CI	aPR, 95%CI	uPR, 95%CI	aPR, 95%CI
**Demographic factors**						
**Gender**						
Male	1.04(0.9-1.2)		0.94(0.85-1.04)		1.1(0.9-1.3)	
Female	Ref		Ref		Ref	Ref
**Age group**						
18-30yrs	1.5(1.2-1.8)**	1.1(0.8-1.4)	1.2(1.03-1.4)*	1.1(0.9-1.2)	0.7(0.6-0.9)**	0.9(0.7-1.2)
31-40yrs	1.3(1.01-1.6)*	1.03(0.8-1.3)	1.1(0.9-1.2)	1.0(0.8-1.1)	0.9(0.7-1.1)	0.9(0.8-1.2)
41-50yrs	1.2(1.0-1.6)	1.04(0.8-1.3)	1.04(0.9-1.2)	1.0(0.8-1.14)	0.8(0.7-1.02)	0.8(0.7-1.1)
>50yrs	Ref	Ref	Ref	Ref	Ref	Ref
**Occupation**						
Unemployed	1.03(0.8-1.4)		1.1(0.9-1.3)		1.2(0.9-1.6)	1.2(0.9-1.6)
Formal employment	0.8(0.6-1.1)		1.0(0.8-1.2)		1.3(1.0-1.7)	1.3(0.9-1.7)
Student	1.1(0.8-1.6)		1.1(0.9-1.4)		0.9(0.6-1.4)	1.2(0.8-1.9)
Retired	Ref		Ref		Ref	Ref
**Marital status**						
Single	1.3(1.1-1.6)**	1.1(0.9-1.3)	1.1(0.96-1.2)		0.8(0.6-0.9)**	0.9(0.8-1.2)
Married	Ref	Ref	Ref		Ref	Ref
Others	1.4(1.14-1.7)**	1.0(0.8-1.3)	1.1(0.9-1.2)		0.7(0.6-0.9)**	1.01(0.8-1.3)
**Education level**						
None/Primary education	1.3(1.01-1.6)*	1.0(0.8-1.2)	1.1(0.9-1.2)		0.8(0.6-0.9)**	0.9(0.7-1.2)
Secondary education	1.3(1.1-1.6)**	1.1(0.9-1.3)	1.03(0.9-1.2)		0.8(0.6-0.9**	0.9(0.7-1.1)
College and above	Ref	Ref	Ref		Ref	Ref
**Source of Information for COVID-19 vaccine**						
**Official government and international websites and media**						
No	0.9(0.8-1.1)		0.9(0.8-0.95)**	0.96(0.86-1.1)	0.9(0.8-1.1)	
Yes	Ref		Ref	Ref	Ref	Ref
**News media, e.g., TVs, radios, newspaper**						
No	1.0(0.8-1.1)		1.04(0.9-1.2)		1.0(0.9-1.2)	
Yes	Ref	Ref	Ref		Ref	Ref
**Social media, e.g., WhatsApp, Facebook, Instagram, and Twitter**						
No	0.6(0.5-0.7)***	0.8(0.7-0.9)**	0.9(0.8-0.95)**	0.89(0.81-0.99)*	1.1(0.97-1.3)	
Yes	Ref	Ref	Ref	Ref	Ref	Ref
**Medical journals**						
No	0.7(0.6-0.8)***	0.8(0.7-1.02)	0.9(0.8-1.03)		1.1(0.86-1.4)	
Yes	Ref	Ref	Ref		Ref	Ref
**Neighbors, friends, and family**						
No	0.54(0.46-0.63)***	0.82(0.7-0.96)*	0.9(0.9-0.95)**	1.1(0.98-1.2)	2.0(1.6-2.3)***	1.5(1.2-1.8)***
Yes	Ref	Ref	Ref	Ref	Ref	Ref
**Social gatherings**						
No	0.7(0.6-0.8)***	1.1(1.0-1.3)	0.7(0.6-0.8)***	0.8(0.75-0.94)**	1.6(1.3-1.9)***	0.9(0.7-1.1)
Yes	Ref	Ref	Ref	Ref	Ref	Ref
**Type of study community**						
Vaccination services seekers	Ref	Ref	Ref	Ref	Ref	Ref
Medical care services seekers	5.3(3.9-7.2)***	4.8(3.4-6.7)***	1.7(1.5-1.9)***	1.6(1.4-1.8)***	0.4(0.350.48)***	0.5(0.4-0.6)***

Adjusted prevalence ratio (APR), confidence interval (CI), P-values: *P-value<0.05, **<0.01 and ***<0.001

**Figure 3:**
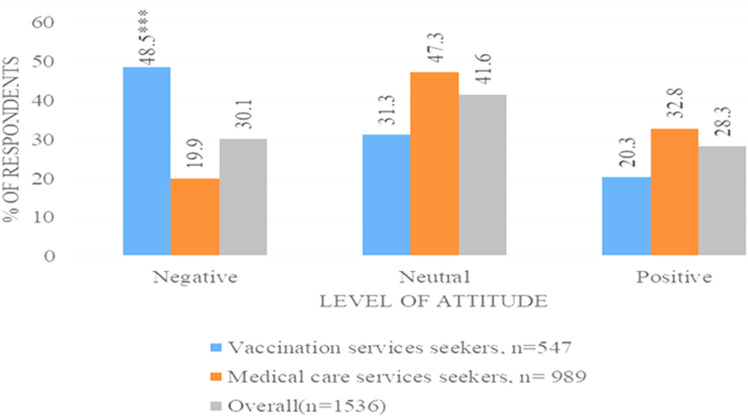
proportion of respondents with negative, neutral, and positive attitude levels towards COVID-19 vaccine by types of community (n=1536)

**Attitudes towards the COVID-19 vaccine:** Figure 3 shows the proportions of participants with different attitudes, 41.6%) of the overall participants had a neutral attitude towards the vaccines followed by 30.1% with a negative attitude and 28.3% with a positive attitude. There was more negative attitude observed within those who came to be vaccinated (48.5%; p-value <0.001) compared to the non-vaccine seekers (19.9%). Table 3 highlights the questions asked to respondents and overall, 73.8% of participants said that they would recommend the vaccines to others. Less than half (47.9%) of all the participants were worried about getting the vaccine and 74.1% had no concerns. Participants who did not gather information from neighbors, friends, and family were found to be 50% less likely to have a negative attitude compared to those who did. Results from the adjusted analysis showed that medical care services seekers aPR=0.5 (95%CI: 0.4-0.6) were less likely to have negative while those who received vaccine information from neighbor/friends aPR=1.5 (95%CI: 1.2-1.8) were more likely to be found with negative attitudes towards vaccines.

**Table 3:** proportion of respondents with positive perception and attitude towards and those with correct knowledge of COVID-19 vaccine by type of community (n=1536)

Statement	Vaccination community, n=547(%)	Non-vaccination community, n= 989(%)	Overall, n=1536(%)	P-value
**Perception**				
Is the COVID-19 vaccine safe? (Best response: It is safe and without side effects or it is safe with some side effects)	409(74.8)	645(65.2)	1054(68.6)	<0.001
How important do you perceive the COVID-19 vaccine to be? (Best response: very important)	500(91.4)	566(57.2)	1066(69.0)	<0.001
How important do you think that everyone in the community should get the COVID-19 vaccine? (Best response: very important)	500(91.4)	539(54.5)	1039(67.6)	<0.001
**Attitude**				
Would you recommend others to receive the vaccine? (Best responses: yes)	489(89.4)	645(65.2)	1134(73.8)	<0.001
Are you worried or anxious about receiving COVID-19 vaccine? (Best response: no)	222(40.6)	514(52.0)	736(47.9)	<0.001
Do you have any concerns about receiving the COVID-19 vaccine? (Best response: no)	202(36.9)	936(94.6)	1138(74.1)	<0.001
**Knowledge**				
Can the COVID-19 vaccine protect you from getting COVID-19? (Best response: yes)	193(35.2)	498(50.4)	691(45.0)	<0.001
Can you get COVID-19 even after taking the COVID-19 vaccine? (Best response: yes)	453(82.8)	469(47.4)	922(60.0)	<0.001
Can the COVID-19 vaccine be given even if you have a previous history of COVID-19 infection? (Best response: yes)	273(49.9)	420(42.5)	693(45.1)	0.005
The best preventive measure for COVID-19 is getting vaccinated. (Best response: yes)	276(50.5)	395(39.9)	671(43.7)	<0.001
Do you believe that the way to overcome the COVID-19 pandemic is by providing mass vaccination? (Best response: yes)	426(77.9)	473(47.8)	899(58.5)	<0.001

**Figure 4:**
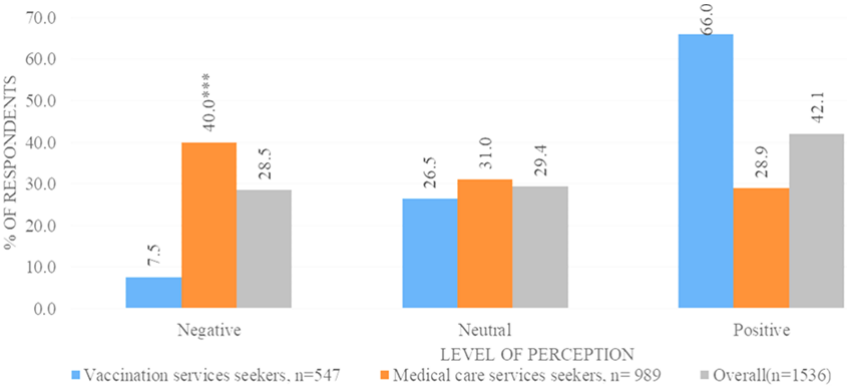
proportion of respondents with negative, neutral, and positive perceptions towards COVID-19 vaccines by type of community (n=1536)

**Perception towards the COVID-19 vaccine:** vaccine seekers (66.0%) exhibited a positive perception towards the COVID-19 vaccines as compared to their counterparts (Figure 4). They responded positively to all questions assessing perception (Table 3). Types of sources of information such as social media, medical journals, neighbors and family, and social gatherings are all significantly associated with negative perceptions of vaccines. Adjusted analysis shows that medical care services seekers were more likely to have a negative perception towards vaccines as compared to vaccine seekers aPR= 4.8 (95%CI: 3.4-6.7), those who did not get information from social media aPR= 0.80 (95%CI: 0.70-0.90) and neighbor/friends aPR= 0.82 (95%CI: 0.70-0.96) were less likely to have a negative perception towards vaccines.

## Discussion

This paper reports findings from a study that compares the knowledge, attitude, and perceptions of two groups who attended SHMH. Findings from this study reflect several factors influencing KAP amongst individuals regarding the COVID-19 vaccines. Vaccine seekers were mostly found to be over the age of 50 years compared to the non-vaccine seekers This is in keeping with several studies where vaccine recipients or those willing to be vaccinated are elderly and have other co-morbidities [17-19]. Another reason for elderly people willing to be vaccinated was that they considered it to be their societal responsibility [20]. The fact that we observed a similar trend informs that the elderly population whether with or without comorbidities are more cautious and worried about contracting the COVID-19 virus and suffering from its consequences. On the other hand, younger individuals between 18-30 years (youths) were highly likely to have low knowledge, negative perceptions, and negative attitudes. This could be attributed to the fact that because they are already aversive to the vaccines, they would not like to know more about it and thus are poorly informed which leads to low knowledge. Our study reveals the knowledge gap among youths, and their acceptance of the vaccine, thus more efforts to engage youths in health education forums in disseminating appropriate information should be made as they are the most populated group in the country and are the most internet users in this era [10].

The most common source of information on COVID-19 vaccines amongst our study population was news media including TV, radio channels, and newspapers contrary to other studies where social media was the most common source [17,21]. However, a study by Chiara Reno on sources of information for COVID vaccines displayed similar results to ours where news media including TV, radio, and newspapers were the most common sources with lesser hesitancy to the vaccine compared to social media users [22]. Different studies have identified the role of social media in promoting or demoting the uptake of vaccines. Theophilus Acheampong revealed a strong positive association between social media use and vaccine hesitancy which has been displayed by our study participants [18]. The rapid growth of communication technology in our country and other African countries is likely to influence communication and news spread in the future as seen in the Western countries.

In our study, the vaccine seekers vaccine community exhibited higher levels of knowledge compared to the non-vaccine seekers community albeit both groups had low levels of knowledge. A low level of knowledge regarding the vaccines has been demonstrated in several studies which have been associated with obtaining information on the vaccine from other sources than news media such as social gatherings, neighbors and friends, and social media including Facebook, WhatsApp, or the internet [16,23]. Our study also showed an association of low knowledge with sources of information, and this could be attributed to the fact that the vaccine seekers actively sought information from more reliable and renowned resources in our setup. Social gatherings and friends can provide information that is their own perspectives or cultural beliefs which hinder the relay of correct information. Similarly, social media platforms may contain fabricated information.

An alarming observation was that vaccine seekers had a higher proportion of individuals with negative attitudes towards the vaccine. This was reflected in their responses to questions on concerns about getting the vaccine. The negative attitude was mostly observed in elderly people aged 50 years and above and could be due to fear of adverse effects. Highly educated people with college degrees also had negative attitudes contrary to findings in Malaysia where the younger educated population was more willing and accepting to get vaccinated [16]. These could be individuals who needed vaccination services in quest of their employers for job security, traveling purposes, or influence by family members or close friends.

Despite the negative attitude of the participants who came to the hospital for the COVID-19 vaccine, they exhibited positive perceptions of the vaccines. Questions assessing perception targeted the safety of the vaccine including side effects, and its importance for oneself and the community in the prevention of infection acquisition. All the sources of information except for news media were associated with negative perceptions in vaccine seekers. These different sources could be misleading and hamper people´s decision-making. Mohamed *et al*. report people are worrisome about the scary information circulating on social media regarding vaccines [16]. A study by John Demuyakor confirmed the same by identifying that a lot of negative perceptions were associated with the use of social media such as Facebook, Twitter, WhatsApp, and YouTube [24]. Unmarried individuals in our study were noted to have negative perceptions of the vaccine. This could partly be attributed to the fact that they don´t have any family responsibilities and would rather not take the vaccine instead of taking it and bearing other unwanted outcomes whilst they may just refrain from contracting the disease naturally. Younger people and those with lower levels of knowledge i.e., secondary and below exhibited negative perceptions of the vaccine, this could be due to being exposed to vast information but not being able to make an evidence-based decision.

## Conclusion

This study identified that non-news media has been linked with negative attitudes and perceptions as well as low levels of knowledge on COVID-19 vaccines in this study population. News media plays a crucial role in people's decision-making process as it influences their thoughts and behaviors. However, collateral damage occurs mostly when information contamination takes place through peers or any other unreliable source of information. We recommend interventions to improve knowledge, attitude, and perception towards vaccines and modulation of sources of vaccine information for improved uptake of vaccines.

### What is known about this topic

- Vaccine hesitancy is a global concern particularly in the COVID-19 pandemic, posing challenges to vaccination efforts globally;
- High level of misconception surrounding COVID-19 vaccine uptake contributing to vaccine hesitancy;
- Impact of vaccine hesitancy on public health including prolongation of the COVID-19 pandemic.

### What this study adds

- The study identifies specific sources of vaccine information and the status of being a non-vaccine seeker as significant predictors of low knowledge, negative attitudes, and perceptions toward COVID-19 vaccines;
- Evidence from a region where unique cultural, social, and economic factors may influence vaccine hesitancy;
- Underscores the need for comprehensive public health campaigns and education initiatives debunking.
